# Automatic data extraction to support meta-analysis statistical analysis: a case study on breast cancer

**DOI:** 10.1186/s12911-022-01897-4

**Published:** 2022-06-18

**Authors:** Faith Wavinya Mutinda, Kongmeng Liew, Shuntaro Yada, Shoko Wakamiya, Eiji Aramaki

**Affiliations:** Graduate School of Science and Technology, Nara Institute of Science and Technology, Nara, Japan

**Keywords:** Automatic meta-analysis, Natural language processing (NLP), Automatic data extraction, Named entity recognition (NER), Evidence-based medicine

## Abstract

**Background:**

Meta-analyses aggregate results of different clinical studies to assess the effectiveness of a treatment. Despite their importance, meta-analyses are time-consuming and labor-intensive as they involve reading hundreds of research articles and extracting data. The number of research articles is increasing rapidly and most meta-analyses are outdated shortly after publication as new evidence has not been included. Automatic extraction of data from research articles can expedite the meta-analysis process and allow for automatic updates when new results become available. In this study, we propose a system for automatically extracting data from research abstracts and performing statistical analysis.

**Materials and methods:**

Our corpus consists of 1011 PubMed abstracts of breast cancer randomized controlled trials annotated with the core elements of clinical trials: Participants, Intervention, Control, and Outcomes (PICO). We proposed a BERT-based named entity recognition (NER) model to identify PICO information from research abstracts. After extracting the PICO information, we parse numeric outcomes to identify the number of patients having certain outcomes for statistical analysis.

**Results:**

The NER model extracted PICO elements with relatively high accuracy, achieving F1-scores greater than 0.80 in most entities. We assessed the performance of the proposed system by reproducing the results of an existing meta-analysis. The data extraction step achieved high accuracy, however the statistical analysis step achieved low performance because abstracts sometimes lack all the required information.

**Conclusion:**

We proposed a system for automatically extracting data from research abstracts and performing statistical analysis. We evaluated the performance of the system by reproducing an existing meta-analysis and the system achieved a relatively good performance, though more substantiation is required.

## Introduction

A meta-analysis is a statistical analysis that combines the results of different studies that are all focused on same disease, treatment, or outcome to determine if a treatment is effective or not. Meta-analyses provide the best form of medical evidence and are an essential tool for enabling evidence-based medicine and clinical and health policy decision-making [1]. Meta-analyses are time-consuming, labor-intensive, and expensive as they require domain experts to manually search, read, and extract data from hundreds of research articles written in unstructured natural language. The number of research articles is increasing exponentially and it is becoming almost impossible to keep up with the high number of biomedical literature [2]. For instance, a recent study showed that more than 50,000 research articles related to the COVID-19 pandemic have been published and more articles are being published every day [3]. The large number of research articles increases the time required to conduct a meta-analysis. Previous research showed that on average it takes about 67 weeks, from registration to publication, to finalize a meta-analysis [4]. This poses a challenge for practitioners in the infectious disease field where informed decisions have to be made promptly. Moreover, most meta-analyses are outdated shortly after publication as they have not incorporated new evidence which might alter the results [5].

Automatic meta-analysis systems have the benefit of reducing the time-taken in conducting a meta-analysis so as to help in timely dissemination of medical evidence and allow for automatic updates when new evidence becomes available. According to surveys on automation of meta-analysis, different strategies for automating the various meta-analysis stages (searching the databases for relevant literature, screening, data extraction, and statistical analysis) have been proposed [6, 7]. Marshall et al. [7] suggests that systems for searching literature, identifying randomized controlled trials (RCTs), and screening articles have attained a good performance and are ready for use. The systems for the data extraction and statistical analysis, on the other hand, are still not readily available.

Techniques for data extraction from research abstracts and full-text articles have been widely studied [6]. Although various methods for extracting different Participants, Intervention, Control, and Outcomes (PICO) information from research articles have been proposed, fewer attempts have been made to extract detailed information for the outcomes, especially numeric texts identifying the number of patients having certain outcomes [8, 9]. Extraction of numeric texts is important for statistical analysis to determine the effectiveness of the intervention. Summerscales et al. [9] used conditional random field-based approach to extract various named entities including treatment groups, group sizes, outcomes, and outcome numbers from research abstracts. Pradhan et al. [8] developed a Web application for extracting data from ClinicalTrials.gov, a clinical trials database. Although ClinicalTrials.gov is an important source of clinical trials data, it has a small number of studies and mainly focuses on clinical trials in the United States [8].

**Fig. 1:**
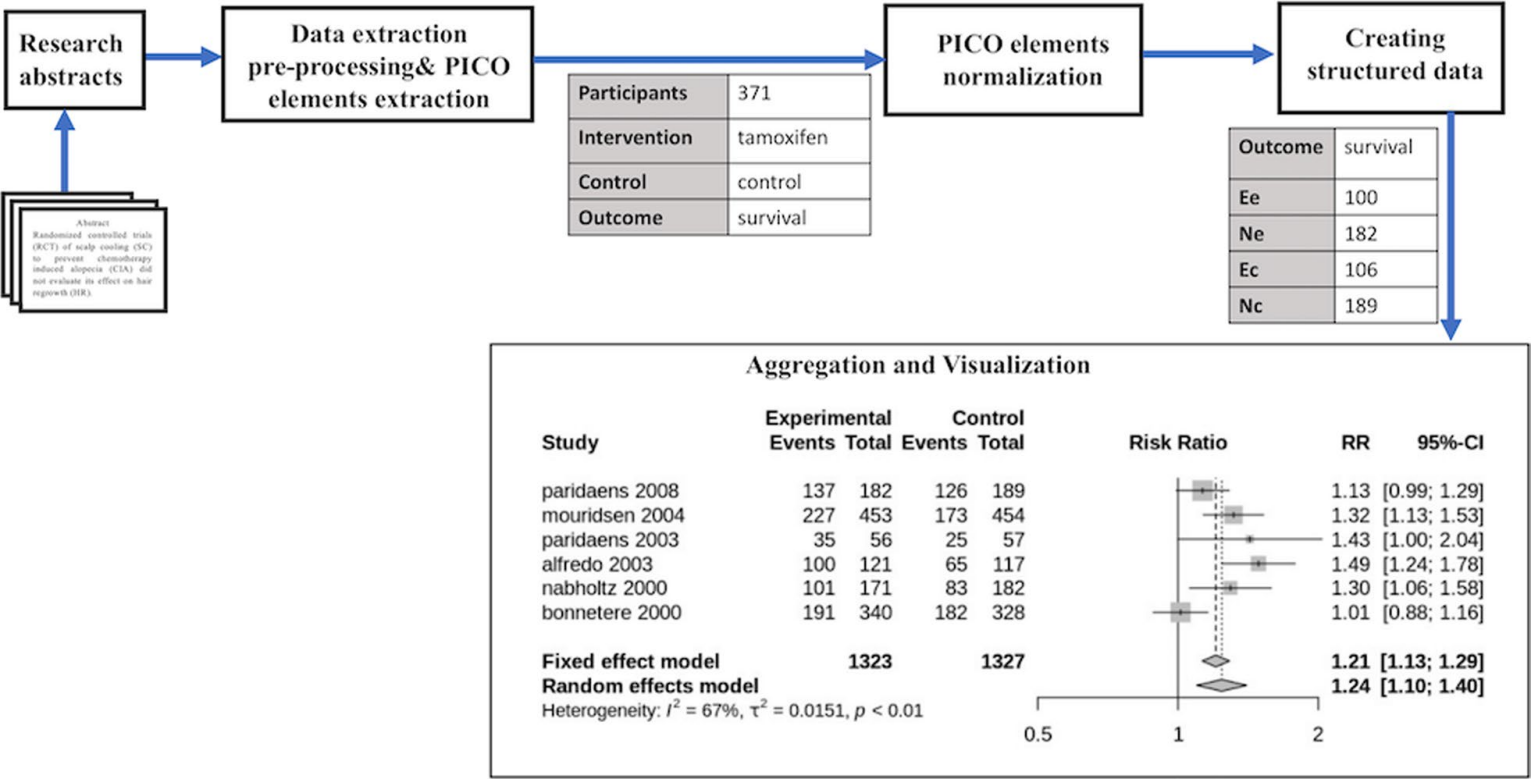
Proposed system architecture

**Fig. 2:**
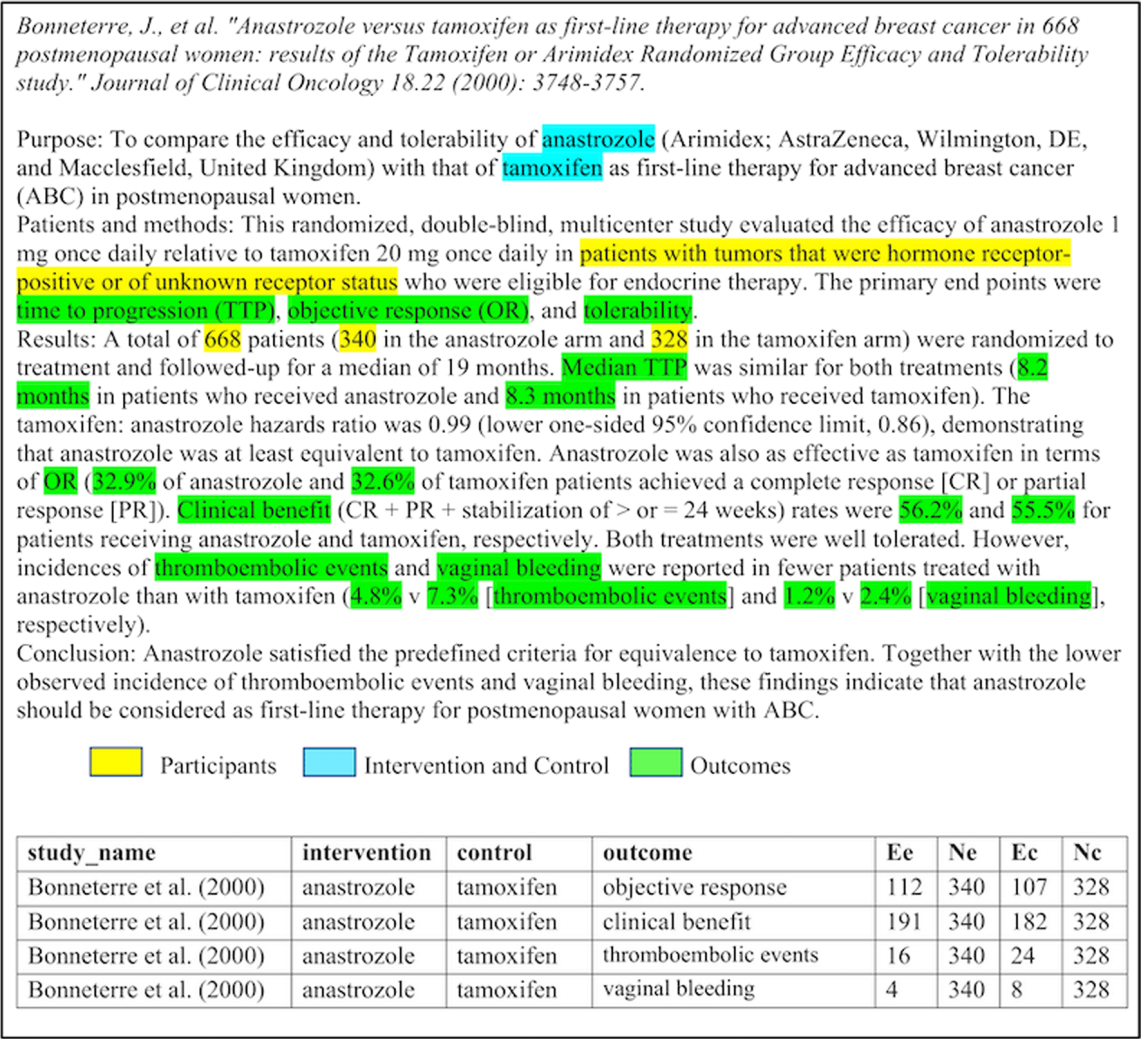
A sample abstract with PICO elements highlighted. The top part shows the abstract while the bottom part shows the PICO elements transformed into a structured format

**Fig. 3:**
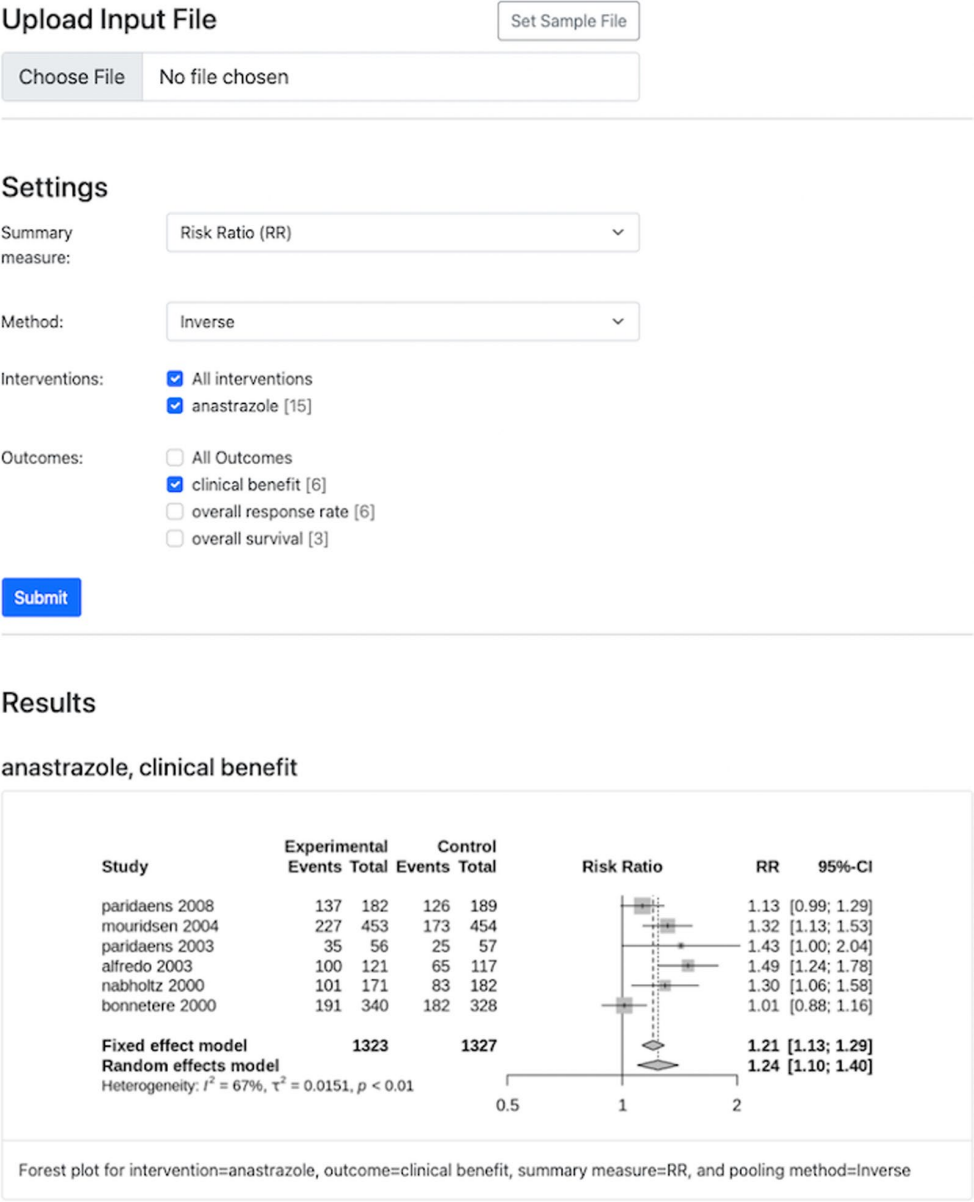
Visualization system interface

The goal of this work is to provide a system that automates data extraction in order to support meta-analysis statistical analysis. We utilize the current state-of-the-art natural language processing (NLP) models to extract PICO information from research abstracts. We use abstracts because they are easily accessible and they provide a concise summary of the full-text article especially the main results. The proposed system (shown in Fig. 1) performs various steps including extracting data from research abstracts, parsing numeric outcomes to identify the number of patients having specific outcomes, converting extracted data into a structured format for statistical analysis, and visualizing the results. We assess the performance of the proposed system by using it to reproduce the results of an existing meta-analysis. The results show potential in automating the tasks and hope to increase interest in research on automating the entire integrated meta-analysis process.

## Materials

The corpus consists of 1011 abstracts of breast cancer randomized controlled extracted from the PubMed.^1^ PubMed is a free search engine that gives access to the MEDLINE database^2^ that indexes abstracts of biomedical and life science research articles. An annotator marked text spans that describe the PICO elements, i.e., Participants (P), Interventions (I), Control (C), and Outcomes (O).

- Participants: text snippets that describe the characteristics of the participants. These include the total number of participants, number of participants in the intervention group, number of participants in the control group, condition, age, ethnicity, location of the study, and eligibility.
- Intervention and Control: text snippets that identify the intervention and control treatments.
- Outcomes: text snippets that identify the outcomes in a study. These include outcomes that were measured, outcome measures, the number of events in the intervention group, and the number of events in the control group.

Outcomes can be classified into binary outcomes and continuous outcomes. Binary outcomes take two values such as the treatment was successful or not. Continuous outcomes take multiple values such as pain which is measured on a numerical scale (pain scores on a scale 0–10). Continuous outcomes are mostly reported as mean, standard deviation, median, or quartiles. The corpus is annotated with different entities to capture the different types of outcomes and their values.

The corpus consists of 1011 manually annotated abstracts. Table 1 shows the frequency of each entity in the corpus. The tags iv, cv, bin, and cont represent intervention group, control group, binary outcome, and continuous outcome respectively. Since binary outcomes numeric texts tend to be absolute values or percentage values, abs and percent are used to represent absolute and percentage values, respectively. Furthermore, for the continuous outcomes we use mean, sd, median, q1, and q3 to represent mean, standard deviation, median, first quartile, and third quartile values, respectively. The corpus is publicly available on our github page.^3^

**Table 1:** Corpus statistics

Category	Sub-category	# tags
Participants	Total-participants	1094
	Intervention-participants	887
	Control-participants	784
	Age	231
	Eligibility	925
	Ethnicity	101
	Condition	327
	Location	186
Intervention	Intervention	1067
Control	Control	979
Outcomes	Outcome	5053
	Outcome-measure	1081
	*Intervention events*	
	bin-abs-iv	556
	bin-percent-iv	1376
	cont-mean-iv	366
	cont-median-iv	270
	cont-sd-iv	129
	cont-q1-iv	4
	cont-q3-iv	4
	*Control events*	
	bin-abs-cv	465
	bin-percent-cv	1148
	cont-mean-cv	327
	cont-median-cv	247
	cont-sd-cv	124
	cont-q1-cv	4
	cont-q3-cv	4

## Methods

### Proposed system architecture

The architecture of the proposed system is shown in Fig. 1. The proposed system consists of five major components: research abstracts, data extraction, PICO elements normalization, creating structured data, and aggregation and visualization. The system input is free-text research abstracts. The research abstracts are passed to the data extraction module for pre-processing and extraction of PICO elements. The extracted PICO elements are then normalized using Unified Medical Language System (UMLS) and dictionary string matching techniques. After normalization, numeric texts are parsed to identify the number of patients having certain outcomes and convert the data into a structured format for statistical analysis. Finally, similar studies (same intervention and same outcome) are grouped together and the results are visualized using forest plots which provide a summary and the extent to which results from different studies overlap.

### Data extraction

#### Pre-processing

The pre-processing step mainly involves acronym expansion. In research articles, acronyms are frequently used to avoid repeating long terms and save space. Even though acronyms simplify writing and reading, they are a major obstacle to natural language text understanding tasks [10]. Generally, acronyms can have multiple common expansions which depend on a particular context. Acronyms commonly occur in the words preceding their first occurrence in parentheses, for example, “Randomized controlled trials (RCT) of scalp cooling (SC) to prevent chemotherapy induced alopecia (CIA)”. In this study, we employ a rule-based method using regular expressions for acronym expansion. The first step in identifying acronyms is to look for terms in parenthesis that are between two and ten characters long. Regular expressions are then used to find expansion candidates in the surrounding text.

#### PICO elements extraction

Data extraction aims to extract PICO elements from research abstracts. This task is formulated as a sequence labelling task, i.e., given a token, classify it as one of pre-defined named entity recognition (NER) tags. As deep learning models have gained a lot of attention in NLP tasks, we adopt Bidirectional Encoder Representations from Transformers (BERT)-based models for this task. BERT has achieved state-of-the-art performance in various NLP tasks including NER and has also proven to be effective for small datasets [11]. BERT is a language model pre-trained on huge amounts of unlabelled data and can be fine-tuned to specific tasks. It uses the encoder structure of the transformer, which is an attention mechanism that learns contextual relations between words (or subwords) in a text.

We chose three pre-trained transformer-based models, i.e., BioBERT [12], BlueBERT [13], and Longformer [14]. BioBERT is pre-trained on different combinations of general and biomedical domain corpora. It is initialized with BERT [11] and further pre-trained on biomedical domain texts (PubMed abstracts and PubMed Central full-text articles). BlueBERT is also initialized with BERT and further pre-trained on PubMed abstracts and clinical notes from MIMIC-III [15]. Longformer is initialized with the RoBERTa model [16] and further pre-trained with books, wikipedia, realnews, and stories.

Traditional transformer-based language models such as BioBERT and BlueBERT cannot attend to long sequences and are limited to a maximum of 512 tokens at a time. This is due to the self-attention operation which grows quadratically with sequence length. Modified transformer models, such as Longformer, have been created to overcome this problem. In Longformer model, the self-attention pattern scales linearly with sequence length enabling it to process longer documents. It can attend to long sequences of up to 4096 tokens, which is 8 times longer than BERT.

### PICO elements normalization

Meta-analysis involves combining similar studies to assess the effectiveness of the intervention (treatment). To automatically group similar studies together and compare them within a meta-study, it is necessary to normalize the extracted PICO elements. We focus on the normalization of the intervention, control, and outcome elements. Our corpus consists of RCTs related to breast cancer, hence all participants are breast cancer patients.

We utilize the UMLS Metathesaurus for the normalization of intervention and control elements. UMLS comprehensively covers most of the interventions and control, especially medications, and hence we did not need to create a normalization dictionary manually. We use MetaMap [17], which is a state-of-the-art NLP tool that maps biomedical text to concepts in the UMLS Metathesaurus. For each text, MetaMap splits the text into phrases and identifies possible mappings for each phrase based on lexical look-up and variants.

A dictionary-based approach was employed for outcome normalization. We extracted all the outcomes from the corpus and manually created a dictionary of the outcomes and their normalizations. For example, pain, breast pain, less pain, and mild pain are all normalized to pain. After creating the dictionary in this manner, we use dictionary string matching techniques to match outcomes and their normalized versions.

The task of matching an outcome with its normalization is defined as; given a predefined set of normalized outcomes *N*, and an input string *o* (outcome), find normalized outcome $n \in N$ that is most similar to *o*. For this task, we utilize a technique that combines Term-Frequency Inverse Document Frequency (TF-IDF), n-grams, and cosine similarity. TF-IDF creates features from text by multiplying the frequency of a term in a document (term frequency) by the importance (inverse document frequency) of the term in the entire corpus. In TF-IDF, usually the term is a word, but depending on the corpus, n-grams have been shown to achieve high performance. For each outcome, we represent the outcome as a vector using TF-IDF and calculate the cosine similarity between the outcome vector and the normalized outcomes vectors and select the normalized outcome with the highest cosine similarity score.

Even though BERT-based models are currently widely used for NLP tasks we utilized a traditional string matching approach for outcome normalization. The current corpus contains many different outcomes which vary greatly with some occurring frequently and others occurring less frequently. Although the BERT models achieve high performance for the outcomes with high frequency, they fail for the outcomes with less frequency. Therefore, we adopted the approach of TF-IDF with cosine similarity, which achieves relatively good performance for both high-frequency and low-frequency outcomes.

### Outcome event matching and creating structured data

Once PICO elements are extracted and normalized, studies with the same intervention and outcome are pooled together so as to compute the overall effect of the intervention. Before calculating the overall effect of the intervention, each study’s treatment effect is determined first. The effect is usually calculated using summary statistics such as risk ratio, odds ratio, or risk difference. In this study, the extracted and normalized PICO elements are converted into a structured format as shown in Fig. 2. To compute the summary statistics, for each outcome four values are required, i.e., *Ee*, *Ne*, *Ec*, and *Nc*. *Ee* is the number of participants in the intervention group that demonstrated effect of the treatment (intervention events), *Ne* is the total number of participants in the intervention group, *Ec* is the number of participants in the control group that demonstrated effect of the treatment (control events), and *Nc* is the total number of participants in the control group. The summary statistics (risk ratio, odds ratio, and risk difference) used in this study are intended for binary outcomes. *Ee* and *Ec* are absolute values that correspond to bin-abs-iv and bin-abs-cv respectively (Table 1). *Ee* and *Ec* can also be calculated from bin-percent-iv and bin-percent-cv as explained in an example further down.

Extraction of the number of participants having certain outcomes is challenging because of lack of uniformity in reporting of results in different articles. We use a rule-based approach for this task and assume that an outcome and its events are reported within the same sentence. If only one outcome is present in a sentence, we assume that the intervention and control events reported in that sentence belong to that outcome. If two or more outcomes are present in a sentence, the first occurrence of intervention events and control events are assigned to the first outcome, the second occurrence of intervention and control events are assigned to the second outcome, and so on. For example, “Overall survival (100% treated, 90.6% controls at 5 years) and disease-free survival (96.2% treated, 86.8% controls at 5 years) were not significantly different in the 2 groups”, we extract (outcome: overall survival, intervention events: 100%, control events: 90.6%) and (outcome: disease-free survival, intervention events: 96.2%, control events: 86.8%). In this example, only percentage values are reported and hence we require knowledge of the number of participants in the intervention and control groups to calculate the absolute values (*Ee* and *Ec*). In some studies, the number of participants in the intervention and control groups (*Ne* and *Nc*) are reported in a different sentence within the abstract (as shown in the sample abstract in Fig. 2) while in other studies they are not reported at all. In the rule-based approach, if the number of participants are not mentioned in the outcome sentence, we check if they are mentioned in the other sentences. Moreover, in some studies words instead of numbers are used, for instance, “Sixty-three percent achieved a complete response ...”, and hence we need to convert the words to numbers. Once the abstracts have been processed in this manner, we get structured data as shown in the bottom part of Fig. 2.

### Meta-analysis results visualization system

We developed a web-based visualization system^4^ for visualizing meta-analysis results. The system was developed using Python and R. R is a powerful and flexible tool that is commonly used when conducting meta-analyses. The calculations of summary statistics were implemented using meta [18], which is an R package commonly used when conducting standard meta-analysis. The results are visualized using forest plots which provide a summary and the extent to which results from different studies overlap. In the forest plot, the effect size of each study is shown and the average effect is shown at the bottom of the plot. Also, in the forest plot, each study is represented by a square whose area represents the weight of the study in the meta-analysis and horizontal line (95% confidence interval).

When using the visualization system, shown in Fig. 3, a user first uploads a csv file. The file must contain columns for study_name, intervention, control, outcome, *Ee*, *Ne*, *Ec*, and *Nc* as shown in the bottom part of Fig. 2. After uploading the file, the user then selects a summary measure and a method for pooling the studies. The available summary measures include risk ratio, odds ratio, and risk difference which are commonly used for binary outcomes. The available pooling methods include inverse variance (Inverse), Mantel-Haenszel (MH), Peto, generalised linear mixed model (GLMM), and sample size method (SSW). For risk ratio and risk difference, only the Inverse or MH pooling methods are used. For odds ratio, inverse, MH, Peto, GLMM, or SSW pooling methods are used. In addition, the user selects the interventions and outcomes for which they would like the results to be visualized. The system groups together similar studies depending on the selected intervention(s) and outcome(s), computes the summary statistics, and returns forest plots. Each forest plot is a summary of studies with the same intervention and the same outcome.

## Results and discussion

### Experimental settings

Our corpus consists of 1011 PubMed abstracts annotated with PICO elements. The frequency of the elements is shown in Table 1. The dataset was split into 80% training set and 20% test set. We developed BERT-based models for data extraction (NER) and compared the performance of general-purpose (Longformer) and biomedical domain (BioBERT, BlueBERT) BERT models. The BioBERT and BlueBERT models cannot attend to sequences longer than 512 tokens (as discussed in the “PICO elements extraction” section). BERT uses WordPiece tokenization and a word can be broken down into more than one sub-words. In the corpus, some abstracts were found to have more than 512 tokens. The default strategy for the BioBERT and BlueBERT models is to truncate long sequences and ignore the tokens after the maximum number is reached. Since truncation leads to loss of information, we split sequences longer than the maximum length into multiple chunks so as to preserve all the information. The split was done in a sentence-wise manner, i.e., if the number of tokens in an abstract is more than 512, we split the abstract into individual sentences, then split the sentences into two halves to create two almost equal chunks. If the number of tokens is greater than 1024, the abstracts are split into three chunks and so on.

In the experiments, we followed the standard pre-trained BERT models for sequence classification. The pre-trained models were fine-tuned on our corpus. The fine-tuning was done by setting the maximum sequence length to 512 tokens for the BioBERT and BlueBERT models and 4096 tokens for the Longformer model. The number of epochs was set to 10, batch size was set to 2, and the learning rate was set to 2e-5 for the BioBERT model and 5e-5 for BlueBERT and Longformer models.

### Data extraction results

The performance of the NER model was evaluated using Precision, Recall, and F1 score in the test set and the results are shown in Table 2. BioBERT_split and BlueBERT_split are the model results where sequences longer than 512 tokens were split into multiple chunks. The Longformer model did not require splitting of abstracts because the maximum sequence length for Longformer is 4096 tokens and there were no abstracts with tokens exceeding the maximum number.

**Table 2:** NER models results

(a) BioBERT model results						
	BioBERT			BioBERT_split		
Sub-category	Precision	Recall	F1	Precision	Recall	F1
Total-participants	0.95	**0**.**95**	**0**.**95**	0.94	0.94	0.94
Intervention-participants	**0**.**80**	0.91	**0**.**85**	0.78	**0**.**93**	**0**.**85**
Control-participants	0.87	**0**.**91**	**0**.**89**	0.85	**0**.**91**	0.88
Age	0.66	0.97	0.79	0.66	0.96	0.78
Eligibility	0.75	0.77	0.76	0.77	0.74	0.76
Ethnicity	0.82	0.89	0.86	0.82	**0**.**96**	**0**.**88**
Condition	0.86	**0**.**81**	**0**.**84**	0.84	0.75	0.79
Location	0.75	**0**.**85**	0.80	0.73	0.81	0.77
Intervention	0.85	0.82	0.84	0.85	0.82	0.84
Control	0.78	0.80	0.79	0.77	0.76	0.77
Outcome	0.82	0.81	0.81	0.84	0.80	0.82
Outcome-measure	0.79	0.90	0.84	0.81	0.88	0.84
bin-abs-iv	0.75	0.78	0.77	0.81	0.78	0.79
bin-abs-cv	0.79	**0**.**87**	0.83	0.77	0.80	0.79
bin-percent-iv	**0**.**87**	0.88	0.87	0.83	0.86	0.84
bin-percent-cv	**0**.**88**	**0**.**90**	**0**.**89**	0.87	0.82	0.84
cont-mean-iv	0.78	**0**.**90**	0.83	0.80	0.86	0.83
cont-mean-cv	**0**.**86**	0.86	**0**.**86**	0.81	0.84	0.83
cont-median-iv	**0**.**70**	0.80	0.75	**0**.**70**	**0**.**86**	**0**.**78**
cont-median-cv	0.76	**0**.**81**	**0**.**78**	**0**.**83**	0.74	**0**.**78**
cont-sd-iv	0.68	**0**.**93**	0.79	0.80	0.85	0.82
cont-sd-cv	0.76	0.84	0.80	0.72	0.85	0.78
cont-q1-iv	0.00	0.00	0.00	0.00	0.00	0.00
cont-q1-cv	0.00	0.00	0.00	0.00	0.00	0.00
cont-q3-iv	0.00	0.00	0.00	0.00	0.00	0.00
cont-q3-cv	0.00	0.00	0.00	0.00	0.00	0.00

(b) BlueBERT model results						
	BlueBERT			BlueBERT_split		
Sub-category	Precision	Recall	F1	Precision	Recall	F1
Total-participants	0.94	0.91	0.92	0.95	0.92	0.94
Intervention-participants	0.72	0.90	0.80	0.73	0.91	0.81
Control-participants	0.81	0.85	0.83	0.79	0.89	0.84
Age	0.67	0.97	0.79	0.66	0.97	0.79
Eligibility	0.73	0.74	0.73	0.73	0.70	0.72
Ethnicity	0.90	0.72	0.80	**0**.**91**	0.78	0.84
Condition	**0**.**90**	0.70	0.79	0.82	0.77	0.79
Location	0.77	0.67	0.71	0.76	0.76	0.76
Intervention	0.80	0.81	0.81	0.84	0.83	0.83
Control	0.72	0.68	0.70	0.78	0.71	0.74
Outcome	0.81	0.79	0.80	0.81	0.80	0.80
Outcome-measure	0.73	0.84	0.78	0.76	0.86	0.81
bin-abs-iv	0.77	0.75	0.76	0.67	0.76	0.71
bin-abs-cv	0.75	0.79	0.77	0.72	0.84	0.78
bin-percent-iv	0.74	0.85	0.79	0.79	0.81	0.80
bin-percent-cv	0.83	0.73	0.78	0.82	0.79	0.80
cont-mean-iv	0.72	0.74	0.73	0.61	0.81	0.69
cont-mean-cv	0.77	0.74	0.75	0.73	0.76	0.74
cont-median-iv	0.65	0.78	0.71	0.67	0.62	0.64
cont-median-cv	0.80	0.66	0.72	0.75	0.66	0.70
cont-sd-iv	0.62	0.68	0.65	0.59	0.60	0.59
cont-sd-cv	0.67	0.68	0.67	0.56	0.70	0.63
cont-q1-iv	0.00	0.00	0.00	0.00	0.00	0.00
cont-q1-cv	0.00	0.00	0.00	0.00	0.00	0.00
cont-q3-iv	0.00	0.00	0.00	0.00	0.00	0.00
cont-q3-cv	0.00	0.00	0.00	0.00	0.00	0.00

(c) Longformer model results						
Sub-category	Precision	Recall	F1			
Total-participants	**0**.**96**	0.94	**0**.**95**			
Intervention-participants	0.79	0.92	**0**.**85**			
Control-participants	**0**.**89**	0.89	**0**.**89**			
Age	**0**.**78**	**0**.**98**	**0**.**87**			
Eligibility	**0**.**89**	**0**.**86**	**0**.**88**			
Ethnicity	0.75	0.83	0.78			
Condition	0.83	0.79	0.81			
Location	**0**.**91**	0.79	**0**.**85**			
Intervention	**0**.**86**	**0**.**85**	**0**.**86**			
Control	**0**.**81**	**0**.**86**	**0**.**83**			
Outcome	**0**.**85**	**0**.**86**	**0**.**86**			
Outcome-measure	**0**.**85**	**0**.**95**	**0**.**90**			
bin-abs-iv	**0**.**83**	**0**.**83**	**0**.**83**			
bin-abs-cv	**0**.**8**4	0.85	**0**.**84**			
bin-percent-iv	0.85	**0**.**90**	**0**.**88**			
bin-percent-cv	**0**.**88**	0.85	0.87			
cont-mean-iv	**0**.**85**	0.87	**0**.**86**			
cont-mean-cv	0.78	**0**.**91**	0.84			
cont-median-iv	0.65	0.76	0.70			
cont-median-cv	0.75	0.76	0.75			
cont-sd-iv	**0**.**83**	0.86	**0**.**85**			
cont-sd-cv	**0**.**77**	**0**.**92**	**0**.**84**			
cont-q1-iv	0.00	0.00	0.00			
cont-q1-cv	0.00	0.00	0.00			
cont-q3-iv	0.00	0.00	0.00			
cont-q3-cv	0.00	0.00	0.00			

Bold texts represent the best score for each sub-category

The performance was relatively high with sub-categories such as total-participants and outcome-measure achieving F1-scores greater than 0.90. Most of the other sub-categories achieved F1-scores greater than 0.80. F1-score was zero for the entities with lowest frequency such as cont-q1-iv, cont-q1-cv, cont-q3-iv, and cont-q3-cv. In overall, BioBERT and Longformer models achieved the highest performance in almost all of the entities.

The Longformer model, which is a general purpose model, performed well compared to the biomedical domain BERT models (BioBERT and BlueBERT). One likely explanation is that the biomedical domain BERT models have a maximum sequence length of 512 tokens and longer sequences are truncated resulting in loss of important contextual information. The Longformer model has a maximum sequence length of 4096 tokens and could therefore build contextual representation of the entire context.

The splitting of long sequences was expected to increase model performance, however, there was no change in the model performance. This could be attributed to loss of useful contexts caused by splitting. However, in this study it is necessary to extract information from the entire abstract. The default strategy for BERT models is to truncate long texts hence leading to loss of important information. The purpose of splitting the abstracts into multiple chunks was to enable extraction of information from the entire abstracts. Even though splitting the abstracts did not improve the performance, we were able to avoid loss of information due to truncation.

Even though automatic extraction of PICO elements from abstracts has been studied widely, only a few studies have attempted extraction of numeric texts that identify the number of patients experiencing specific outcomes. We developed a rule-based approach (discussed in “Outcome event matching and creating structured data” section) to parse numeric texts to identify the patients having certain outcomes. The rule-based approach was able to extract outcomes and their events from 77% of the outcome sentences in the gold test set. The rule-based approach however cannot extract outcomes and their events in cases where the outcomes and events are reported in different sentences or in studies other than double-arm studies (one intervention group and one control group).

### System evaluation

To evaluate the performance of the proposed system, we selected a published meta-analysis and used our system to reproduce the results. The selected meta-analysis was conducted by Feng et al. [19] and examines the effect of platinum-based neoadjuvant chemotherapy on resectable triple-negative breast cancer patients. The meta-analysis consists of nine studies, Alba et al. [20], Ando et al. [21], Gluz et al. [22], Loibl et al. [23], Sikov et al. [24], Tung et al. [25], Minckwitz et al. [26], Wu et al. [27], and Zhang et al. [28].

The results are shown in Table 3. The NER model successfully extracted data from the abstracts of the nine studies. There was a NER model prediction error in one study as shown in bold underlined text in Table 3. For the study Gluz et al. [22] and pathological complete response outcome, the model misclassified *Ne* as *Nc* and vice-versa. In this study, the *Ee* and *Ec* values were reported as percentage values. The absolute values of *Ee* and *Ec* were therefore calculated based on the *Ne* and *Nc* values (as discussed in “Outcome event matching and creating structured data” section). Since the system extracted *Ne* and *Nc* values were incorrect, the calculated *Ee* and *Ec* values were also incorrect.

**Table 3:** Results of selected meta-analysis

Study	Outcome	Gold values				System extracted values			
		Ee	Ne	Ec	Nc	Ee	Ne	Ec	Nc
Alba et al. [20]	Pathological complete response	14	47	16	46	14	48	16	46
Ando et al. [21]		23	37	10	38	23	37	10	38
Gluz et al. [22]		70	154	52	182	$\underline{\mathbf{44, 30 }}$	$\underline{\mathbf{182 }}$	$\underline{\mathbf{84, 81 }}$	${\underline{\mathbf{154 }}}$
Loibl et al. [23]		92	160	49	158	92, 168	160	49	158
Sikov et al. [24]		60	110	43	105	60%, 59%, 54%	NA	44%, 48%, 41%	NA
Tung et al. [25]		9	40	10	36	18%	NA	26%	NA
Minckwitz et al. [26]		90	158	67	157	129, 84, 45	137, 158	108, 58, 50	136, 157
Wu et al. [27]		24	62	8	63	24	62	8	63
Zhang et al. [28]		18	47	6	44	18	47	6	44
Alba et al. [20]	Objective response rate	36	47	32	46	37	48	32	46
Wu et al. [27]		58	62	46	63	58	62	46	63
Zhang et al. [28]		42	47	34	44	42	47	34	44

Ee is the number of events in the intervention group, Ne is the number of participants in the control group, Ec is the number of events in the control group, and Nc is the number of participants in the control group. NA indicates where the information was not available in the abstract. $\underline{\mathbf{Bold underlined texts }}$ are NER model prediction errors while ${\underline{italic \,underlined \,texts}}$ are values where extra pre-processing was required

Although the NER model had high accuracy, there were other factors that prevented the full reproduction of the meta-analysis. The italic and underlined texts represent studies where extra post-processing steps were required. For instance, for the studies Loibl et al. [23] and Sikov et al. [24], and pathological complete response, the studies have multiple intervention and control groups. The Gluz et al. [22] and Minckwitz et al. [26] studies, for the pathological complete response outcome, the abstracts report results for different sub-groups. The current system considers only double-arm studies (studies with one intervention group and one control group) and does not perform subgroup analysis, and these will be one of our important future works. Moreover, in some studies, the total number of participants in the intervention and control groups (*Ne* and *Nc*) were not reported in the abstracts. The studies where the numbers were not reported are indicated as NA in Table 3. In the Sikov et al. [24] and Tung et al. [25] studies, we were not able to calculate the absolute values for *Ee* and *Ec* because their calculation depends on the *Ne* and *Nc* values which were not reported in the abstracts.

### Error analysis

We performed an error analysis and identified miclassified entities and boundary detection as the major types of errors.

- Misclassified entities: the model detected the correct boundaries for entities but assigned them the wrong classes. For example, the model sometimes misclassified bin-abs-iv as bin-abs-cv and vice versa (as discussed in the “System evaluation” section).
- Boundary detection: this is where the model identifies shorter or longer entities than those marked in the gold set. The boundary detection error was common in the outcome and eligibility entities. Human annotation could contribute to this error, because sometimes it is difficult to decide the start and end spans of some entities.

### Limitations and future work

Our study has several limitations. This study uses abstracts only and as seen in the “System evaluation” section, abstracts sometimes lack information that is present in the full-text document. For instance, a manual check of our corpus found that a significant number of abstracts do not mention the number of participants in the intervention and control groups. This presents a challenge when determining the number of patients having certain outcomes for statistical analysis. We also do not account for participants who drop out of a study and this might affect the final results. For future work, it is important to consider extracting information from full-text articles.

We proposed a rule-based system for matching outcomes and their events (discussed in “Outcome event matching and creating structured data” section). The rule-based approach considers only double-arm studies, i.e., studies with one intervention group and one control group. Single-arm studies and studies with more than multiple intervention or control groups are ignored. In future, it is necessary to explore other approaches such as relation extraction.

In the statistical analysis step, we consider only binary outcomes. The summary statistics (odds ratio, risk ratio, and risk difference) used in our results visualization system are only focused on binary outcomes. Incorporating continuous outcomes and their summary statistics is important future work. Moreover, some meta-analyses perform subgroup analysis where they compare the results of different subgroups of participants either by age or cancer type. Annotation and incorporation of such information is also necessary in future. Finally, we assessed the performance of the proposed system by replicating the results of an existing meta-study. To substantiate the usefulness of the system, it is important to test it on larger and more complex meta-studies.

## Conclusion

In this paper, we proposed a system for automating data extraction to support meta-analysis statistical analysis. Our objective is to provide a system that automates data extraction and statistical analysis, to shorten the time it takes to carry out a meta-analysis and allow for automatic updates when new results becomes available. The proposed system extracts PICO elements from research abstracts, parses numeric outcomes to extract the number of patients experiencing certain outcomes, transforms the extracted information into a structured format, performs statistical analysis, and visualizes the results in forest plots. We evaluated the performance of the system by attempting to reproduce the results of an existing meta-analysis. The system extracted PICO elements from the studies with high accuracy. The statistical analysis step did not perform well owing to lack of some information in the abstracts and lack of uniformity in the research abstracts were some abstracts required extra pre-processing. These results however show that there is potential to automate these tasks and wish to motivate more research towards fully automating the entire meta-analysis process.

## Data Availability

The dataset used in this article can be freely and openly accessed at our github page: https://github.com/sociocom/PICO-Corpus.

## Abbreviations

PICO: Participants, intervention, control, and outcomes
NER: Named entity recognition
NLP: Natural language processing
RCTs: Randomized controlled trials
UMLS: Unified Medical Language System
TF-IDF: Term-frequency inverse document frequency
BERT: Bidirectional encoder representations from transformers
MH: Mantel–Haenszel
GLMM: Generalised linear mixed model
SSW: Sample size method
Ee: Number of events in the intervention group
Ne: Number of participants in the control group
Ec: Number of events in the control group
Nc: Number of participants in the control group

## Footnotes

1 https://www.nlm.nih.gov/bsd/pmresources.html.

2 https://www.nlm.nih.gov/medline/medline_overview.html.

3 https://github.com/sociocom/PICO-Corpus.

4 https://aoi.naist.jp/autometavisualization/.
